# Review of 5-FU resistance mechanisms in colorectal cancer: clinical significance of attenuated on-target effects

**DOI:** 10.20517/cdr.2022.136

**Published:** 2023-04-29

**Authors:** William H. Gmeiner, Charles Chidi Okechukwu

**Affiliations:** ^1^Department of Cancer Biology and Comprehensive Cancer Center, Wake Forest University School of Medicine, Winston-Salem, NC 27157, USA.; ^2^Integrative Physiology and Pharmacology Graduate Program, Institution, Wake Forest University School of Medicine, Winston-Salem, NC 27157, USA.

**Keywords:** Fluoropyrimidine, 5-FU resistance, colorectal cancer, chemotherapy, precision medicine, thymidylate synthase

## Abstract

The emergence of chemoresistant disease during chemotherapy with 5-Fluorouracil-based (5-FU-based) regimens is an important factor in the mortality of metastatic CRC (mCRC). The causes of 5-FU resistance are multi-factorial, and besides DNA mismatch repair deficiency (MMR-D), there are no widely accepted criteria for determining which CRC patients are not likely to be responsive to 5-FU-based therapy. Thus, there is a need to systematically understand the mechanistic basis for 5-FU treatment failure and an urgent need to develop new approaches for circumventing the major causes of 5-FU resistance. In this manuscript, we review mechanisms of 5-FU resistance with an emphasis on: (1) altered anabolic metabolism limiting the formation of the primary active metabolite Fluorodeoxyuridylate (5-Fluoro-2'-deoxyuridine-5'-O-monophosphate; FdUMP); (2) elevated expression or activity of the primary enzymatic target thymidylate synthase (TS); and (3) dysregulated programmed cell death as important causes of 5-FU resistance. Importantly, these causes of 5-FU resistance can potentially be overcome through the use of next-generation fluoropyrimidine (FP) polymers (e.g., CF10) that display reduced dependence on anabolic metabolism and more potent TS inhibitory activity.

## INTRODUCTION

It is estimated that 1.93 million colorectal cancer (CRC) cases will be newly diagnosed in 2022 worldwide, with 0.94 million CRC-caused deaths. According to the American Cancer Society (ACS), CRC is the 2^nd^ most common cause of cancer-related mortality in the Unite States, accounting for ~51,000 deaths annually^[1,2]^. Surgical approaches are the primary treatment modality for limited-stage CRC when there is no evidence of distant metastasis. However, in elderly patients that constitute most new CRC diagnoses, there is an increased risk of post-operative complications^[3]^. Adjuvant chemotherapy with 5-Fluorouracil-based (5-FU-based) combinations reduces the risk of disease recurrence in stage III and high-risk stage II CRC. Chemotherapy with 5-FU-based combinations together with biologics (e.g., bevacizumab or cetuximab) and immunotherapy in some instances are also used to treat metastatic CRC (mCRC)^[4,5]^, which often occurs in liver and is frequently not amenable to surgical resection.

The chemotherapeutic molecule most widely used for CRC treatment is 5-fluorouracil (5-FU), a synthetic fluorinated pyrimidine (FP) analog of uracil that is used to treat > 2 million cancer patients each year worldwide^[6,7]^. In addition to its widespread use for CRC treatment, 5-FU is also widely used to treat pancreatic, stomach, esophageal, breast, and head-and-neck cancer. 5-FU belongs to the antimetabolite class of anti-cancer drugs^[8,9]^,{Chen, 2019 #42}{Chen, 2019 #42}{Chen, 2019 #42}{Chen, 2019 #42} and its activity results from intracellular conversion into active metabolites that interfere in thymidine biosynthesis and affect DNA- and RNA-mediated processes^[10,11]^{Chen, 2019 #42}{Chen, 2019 #43}. The primary molecular target of 5-FU’s anti-cancer activity is thymidylate synthase (TS), which is required for *de novo* thymidylate (thymidine 5’-O-monophosphate) biosynthesis^[12]^. TS is a well-validated target for cancer chemotherapy^[13]^ and aggressive malignant cells are relatively more reliant on *de novo* thymidylate biosynthesis than non-malignant cells that utilize the alternative salvage pathway^[14]^. The importance of targeting TS for 5-FU’s anti-cancer activity is underscored by its invariant clinical use in combination with folinic acid (Leucovorin; LV), a reduced folate co-factor that binds TS in a ternary complex with 5-Fluoro-2’-deoxyuridine-5’-O-monophosphate (FdUMP), the 5-FU metabolite that irreversibly inhibits TS enzymatic activity. TS inhibition depletes cellular stores of thymidylate, resulting in increased misincorporation of 2′-deoxyuridine-5′-triphosphate (dUTP) in DNA. In cells treated with FP drugs, 5-fluoro-2′-deoxyuridine-5′-triphosphate (FdUTP) is also misincorporated into DNA, and this causes Topoisomerase 1 (Top1)-mediated DNA damage^[15]^. The Gmeiner lab has developed FP polymers (e.g., CF10) that directly release FdUMP without a requirement for anabolic metabolism. CF10 inhibits TS at 100-1,000-fold lower concentrations than 5-FU in CRC cells^[16-18]^ and causes extensive Top1-mediated DNA damage to generate increased replication stress, a point of therapeutic vulnerability in CRC cells.

While the anti-cancer activities of 5-FU and other FP drugs are considered to primarily result from TS inhibition and DNA damage, only a relatively small percentage of 5-FU administered to humans is converted to FdUMP and DNA-directed metabolites (< 5%^[19]^. Most 5-FU (~80%) is either degraded in the liver or excreted intact in the urine^[20]^. Among anabolic metabolites, ribonucleotides are produced at approximately 10-fold greater levels than deoxyribonucleotides^[20,21^**^]^**. The importance of RNA-directed metabolites for 5-FU’s anti-cancer activity remains an active area of investigation^[22]^; however, the systemic toxicities associated with RNA-directed metabolites are established and include gastrointestinal tract toxicity^[23,24]^ and immunosuppression^[23,25]^, both of which are alleviated by uridine administration^[26]^ to dilute 5-FU’s effects on RNA-mediated processes. Patients that are deficient in 5-FU catabolism are highly vulnerable to serious systemic toxicities if treated with 5-FU^[27]^. Approximately 5% of the human population display polymorphisms in the gene encoding dihydropyrimidine dehydrogenase (*DPYD*) that catalyzes the initial step in 5-FU degradation and 5-FU use at standard levels is contraindicated in these patients^[28]^.

**Figure 1:**
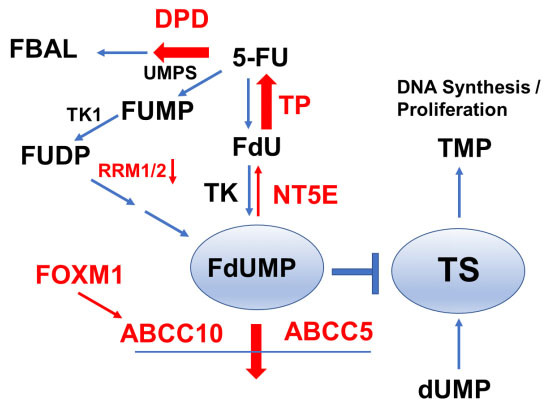
5-FU Resistance can develop through either increased intratumor degradation, decreased anabolic metabolism to FdUMP and/or increased efflux of FdUMP - all of which decrease TS inhibition in tumor cells. 5-FU may be degraded via intratumor DPD, while its anabolic metabolism to FdUMP occurs primarily via UMPS/RRM1/2. Decreased FdUMP levels may result from less efficient conversion through decreased RRM1/2 expression or from increased FdUMP breakdown through elevated expression of NT5E and TP. FdUMP also undergoes efflux from tumor cells by ABC transporters, ABCC10 and ABCC5, that are upregulated upon increased FOXM1 expression in 5-FU-resistant cancer cells. Processes contributing to decreased intra-tumor FdUMP and 5-FU resistance are indicated by red arrows. 5-FU: 5-Fluorouracil; FdUMP: 5-Fluoro-2’-deoxyuridine-5’-O-monophosphate; UMPS: UMP synthase; FBAL: α-fluoro-β-alanine; DPD: dihydropyrimidine Dehydrogenase Deficiency; FUMP: 5-fluorouridine-5'-monophosphate; FUDP: 5-fluorouridine diphospho; FdU: 5-fluoro-2’-deoxyuridine; TK: thymidine kinase; TS: thymidylate synthase; TMP: Trimethylolpropane; RRM: RNA recognition motif; NT5E: ecto-5′-nucleotidase; FOXM1: forkhead box M1; dUMP: 2’-deoxyuridine-5’-O-monophosphate.

**Figure 2:**
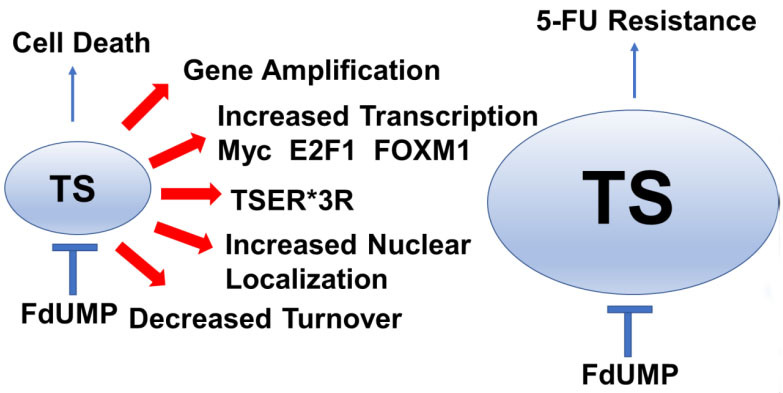
5-FU Resistance develops from processes that increase thymidylate synthase (TS) activity in cancer cells. Increased TS activity can result from multiple processes including gene amplification, increased transcription, TSER*3R polymorphism, increased TS nuclear localization, and decreased TS protein degradation, which are indicated by red arrows. Increased TS activity renders cells 5-FU-resistant because FdUMP levels are insufficient to inhibit all the TS available. 5-FU: 5-Fluorouracil; FdUMP: 5-Fluoro-2’-deoxyuridine-5’-O-monophosphate; FOXM1: forkhead box M1.

5-FU remains a central component of CRC treatment both in the adjuvant setting and in the treatment of mCRC^[4,5]^, which is the cause of cancer-related lethality. While 5-FU is just one component in combination therapy regimens such as FOLFOX and FOLFIRI that combine folinic acid, 5-FU, and either oxaliplatin (FOLFOX) or irinotecan (FOLFIRI), understanding the mechanistic basis for 5-FU resistance can help guide the development of new and more effective therapeutic approaches. FOLFOX or FOLFIRI are frequently used in frontline treatment of mCRC, often in combination with a biologic, such as bevacizumab^[29]^. While current 5-FU-based chemotherapy regimens have contributed to significantly improved survival for mCRC patients (~20 months;^[30]^), 5-year survival remains rare, < 14%, indicating a critical need to understand the mechanistic basis of resistance and develop new strategies to more completely eradicate metastatic disease^[31]^. Innate or acquired resistance remains a prominent cause of treatment failure for patients with metastatic cancer. 5-FU resistance can result from multiple causes; however, a critical review of the literature indicates cancer cells adapt to 5-FU’s cytotoxic effects through: (1) decreasing intracellular FdUMP levels [Figure 1]; (2) elevating activity of the target enzyme, TS; and (3) dysregulating the balance between autophagy and apoptosis to favor cell survival. These endpoints are achieved via multiple mechanisms making overcoming resistance a challenging endeavor. This review focuses on addressing the causes of clinical resistance to 5-FU, considering both clinical data and cellular models of CRC. We review mechanisms by which 5-FU-based therapy fails, intending to provide insight into novel strategies to overcome resistance and improve outcomes beyond the incremental gains achieved in recent years^[32]^.

## CLINICAL DETERMINANTS OF 5-FU RESPONSE IN CRC TREATMENT

The applicability of 5-FU-based chemotherapy for CRC treatment depends upon several factors. For patients with stage III CRC or diagnosed with stage II CRC with risk factors consistent with an elevated likelihood for relapse, 5-FU-based adjuvant chemotherapy is recommended unless tumor biopsy demonstrates high microsatellite instability (MSI-H) or deficiency in DNA mismatch repair (MMR-D). For patients with MSI-High or MMR-D primary CRC tumors, which include familial syndromes such as Lynch syndrome, 5-FU-based regimens are ineffective and testing for MMR-D status prior to treatment is standard care. Testing for MMR-D status is also required for establishing responsiveness to immune checkpoint blockade immunotherapy, which is relatively more effective in CRC patients with high tumor mutational burden associated with MMR-D^[33]^. MSI testing by polymerase chain reaction (PCR) and immunohistochemistry (IHC) is used to establish MMR-D^[34]^. Two antibody IHC testing for MSH6 and PMS2 is used to identify MMR-proficient CRC patients, and if deficiency is suspected, IHC for mutS homolog 2 (MSH2) and mutL homolog 1 (MLH1) are undertaken to establish MMR-D^[35]^. MLH1 promoter methylation testing is done for cases with MLH1-IHC loss.

Relevance of MMR-D for CRC chemotherapy is that, in general, MSI-High and MMR-D in early-stage primary colon cancer confer a good prognosis and NCCN does not recommend adjuvant 5-FU for stage II CRC that is MSI-high. However, the FOLFOX regimen is beneficial in MSI-high stage III^[36]^, and patients with Transforming growth factor-β_RII (_TGF-β_RII_) mutations in particular may be responsive to 5-FU-based therapy^[37]^. The TGF-β pathway also is implicated in drug resistance in pre-clinical studies and specific inhibition of TGF-β_I_ restored the sensitivity of resistant CRC cells to 5-FU^[38]^. Similarly, for patients with mCRC that is MSI-H or MMR-D, alternative frontline therapy to 5-FU-based therapy is implemented, frequently immune checkpoint blockade^[39]^. The mechanism by which deficient MMR renders 5-FU-based regimens ineffective is not definitively known. In principle, MMR may remove 5-FU from DNA and genomic silencing or mutation of MMR genes may increase 5-FU in DNA^[40]^, which could accentuate DNA damaging processes, which include DNA topoisomerase 1 poisoning^[41]^. Alternatively, MMR proficiency may contribute to cancer cell death through the activation of a futile cycling mechanism^[42]^. Our studies indicate MMR status does not significantly affect the response of CRC cells to either 5-FU or CF10^[43]^, indicating the observed clinical cause of MMR dependence may not be directly related to DNA repair. Base excision repair (BER) actively removes 5-FU from DNA in CRC cells^[44,45]^, but BER is not a determinant in 5-FU clinical response. However, CpG island methylator phenotype, and chromosome instability^[46]^, two processes that differentiate the etiology of CRC development, are implicated in 5-FU response^[47,48]^.

## MECHANISMS INCREASING TS TO CAUSE 5-FU RESISTANCE

TS is considered the primary molecular target for the anti-cancer activities of FP drugs (5-FU, capecitabine, and floxuridine). TS also is targeted by anti-folates such as tomudex and raltitrexed^[49]^. The clinical success of these drugs, as well as more recent FP-based combination approaches that target TS such as S1 (tegafur, gimeracil, potassium oxanate)^[50]^ and Lonsurf or TAS-102 (trifluridine, Tiperacil)^[51]^, establishes TS as a central and well-established chemotherapy target^[13]^. The structural basis for TS inhibition by FPs was shown to result from nucleophilic attack by Cys195 at C6 of FdUMP, resulting in irreversible enzyme inhibition via a ternary complex that also includes a reduced folate co-factor^[49]^.

The relationship between TS levels and response to 5-FU, other FPs, or TS inhibitors is complex, in part because while elevated TS levels contribute to resistance (since more FdUMP is required for TS inhibition), but very low TS levels slow cell proliferation, which is necessary for replication-dependent DNA damage. Further, establishing elevated TS as a cause of resistance to 5-FU or other TS-targeted therapeutics is challenging because TS is regulated at multiple levels including through gene amplification, polymorphisms in the promoter, and upregulation of transcription factors that regulate its intratumor expression [Figure 2]. TS levels and activity^[52]^ significantly correlate with response to 5-FU-based treatment and LV enhanced TS inhibition. However, incorporation of 5-FU into either DNA or RNA does not correlate with response to 5-FU^[53]^.

### Transcriptional regulation of TYMS

Transcriptionally, *TYMS* (encoding TS) is regulated by E2F family transcription factors^[54]^ in an S-phase-dependent manner^[55]^. *TYMS* expression is also sensitive to Myc levels and silencing *TYMS* decreases the oncogenic properties of elevated MYC in some cell contexts^[56]^. Recently, an analysis from the Cancer Genome Atlas (TCGA) database revealed lower *TYMS* was associated with better response to FOLFOX/FOLFIRI therapy in mCRC patients and MYC was identified as an upstream controller of genes that regulate response to 5-FU+folate therapy^[57]^. The forkhead transcription factor forkhead box M1 (FOXM1) is regulated by E2F1 and directly upregulates *TYMS* and is responsive to DNA damage. Elevated FOXM1 is a cause of 5-FU resistance through the upregulation of *TYMS*^[58]^, and recent studies indicate targeting FOXM1 can overcome 5-FU resistance^[59]^. Other signaling pathways may upregulate *TYMS* and cause 5-FU resistance, including HSP90/Src^[60]^. Further, *TYMS* is regulated by the MALAT1-miRNA network^[61]^ and other miRNAs that regulate drug resistance^[62]^ and can be used as biomarkers^[63]^.

### Gene amplification of TYMS

The importance of TS gene and protein expression for 5-FU resistance was established in CRC tumors. Responsive patients had significantly lower mean TS protein and gene levels relative to non-responsive patients^[64]^. Further, CRC cells selected for acquired 5-FU resistance displayed elevated TS, which occurred through gene amplification^[65]^. Elevated TS is associated with clinical resistance to 5-FU^[66]^, consistent with TS being the primary molecular target of FPs. Several studies^[67,68]^, including a meta-analysis of 13 studies^[69]^, demonstrated that elevated TS was associated with poor outcomes. However, multiple studies indicate the relationship between TS expression and 5-FU response is complex and may depend on the extent of TS nuclear localization or the expression of other genes, particularly those regulating 5-FU metabolism including dihydropyrimidine dehydrogenase deficiency (DPD) and TP^[66]^. TS undergoes reversible SUMOylation^[70]^ and localizes to the nucleus (nTS) as part of a multi-protein complex that enables efficient *de novo* ddP biosynthesis during S-phase^[71]^. Clinical studies indicate that increased intratumor nuclear localization of TS may be a better indicator of disease aggressiveness than overall TS levels^[72]^. *TYMS* gene amplification is detected in mCRC from patients pre-treated with 5-FU-based chemotherapy and was associated with shorter median survival for patients treated with chemotherapy following surgical resection^[73]^. A summary of studies in which TS gene amplification was implicated with 5-FU resistance is included in Table 1.

**Table 1:** TS Gene Amplification in 5-FU Resistance

**Tissue/cells**	**Frequency/treatment**	**Site**	**Reference**
mCRC	18%	Liver metastases	[73]
mCRC	23%	Liver metastases	[74]
CRC	Increased progression	Colon cancer	[75]
CRC cells	FdU treatment	Colon cancer cells	[76]
CRC cells	5-FU treatment	Colon cancer cells	[65]

TS: thymidylate synthase; mCRC: metastatic colorectal cancer; CRC: colorectal cancer.

### TSER and alternative causes of elevated TS

In addition to *TYMS* gene amplification and increased transcription, at least three other processes are potential factors that could increase TS levels and contribute to 5-FU resistance [Figure 2]: i) TS enhancer region (TSER) polymorphisms; ii) TS translational autoregulation; iii) TS proteasomal degradation. Polymorphisms in the 5′-UTR of *TYMS* contribute to elevated *TYMS* expression in some contexts^[77,78]^. A triple tandem repeat (TSER*3)^[79]^ in the 5′-UTR of the TS gene^[80]^ resulted in elevated *TYMS* expression and 5-FU resistance^[81]^. The clinical significance of TSER genotypes remains largely unproven in CRC; however, prospective selection of patients with gastric cancer have at least one TSER*2 allele favoring lower *TYMS* expression therapy resulted in an encouraging disease control rate for treatment with FOLFOX^[82]^. Further, *TYMS* polymorphisms, together with *KRAS* and *BRAF* mutation status, retrospectively, were associated with reduced relapse in CRC^[83]^. TS also poses a negative autoregulatory function at the translational level by binding to its own mRNA; thus, it prevents the synthesis of functional TS enzyme^[73]^. Autoinhibition of TS protein expression is countered by FdUMP binding and ternary complex formation. At present, there is no evidence that autoregulation of TS by this mechanism contributes to 5-FU resistance. However, another aspect of translation is affected by 5-FU, which is the efficiency and selection of proteins translated by the ribosome^[84]^. TS also undergoes proteasomal degradation and TS levels reflect a dynamic balance of new protein synthesis, dependent upon gene expression and translational efficiency, that is countered by the rate of degradation for expressed protein. A recent study showed that decreased O-GlcNAc transferase (OGT), an enzyme responsible for post-translational modification of multiple proteins including TS, affected TS proteasomal degradation in 5-FU-resistant cells^[85]^.

### Increased TS and intrinsic 5-FU resistance

The elevated expression of TS is commonly accepted as a primary molecular mechanism for acquired 5-FU resistance^[86]^, but it also is important for intrinsic resistance. The stability of the ternary complex is highly dependent on 5,10-methylenetetrahydrofolate (CH_2_THF) levels^[78]^, and lack of CH_2_THF creates an unstable TS: FdUMP binary complex resulting in poor inhibition^[81,86,87]^. Increased TS level prior to 5-FU-based treatments is associated with perturbed folate pools, which cause intrinsic resistance compared to acquired resistance associated with upregulated *TYMS* expression and gene amplification^[73,86]^. These findings suggest that patients with tumors showing TS amplification prior to treatment should not be treated with 5-FU to avoid systemic toxicity without the likelihood of clinical benefit^[73,74]^.

## GENES MODULATING 5-FU METABOLISM

Acquired drug resistance is a principal cause of treatment failure and significantly contributes to cancer-related mortality. In the case of 5-FU, elevated TS is clinically established as a significant cause of drug resistance^[69]^. Still, other reasons have been identified, and prominent among them are alterations in genes that modulate 5-FU metabolism, affecting both its degradation and its conversion to FdUMP, the TS inhibitory metabolite^[88]^ [Figure 1]. A key aspect of 5-FU activity, toxicity, and resistance is mediated by *DPYD*, the gene encoding DPD, the first and rate-limiting step in 5-FU degradation. Atypical 5-FU degradation in liver is associated with serious systemic toxicities^[89]^. In many countries, genetic screening is used to identify CRC patients with *DPYD* polymorphisms associated with decreased DPD activity that result in serious 5-FU toxicities unless the administered dose is reduced from standard dosing^[90]^. Since DPD is not the only potential cause of altered 5-FU toxicity or sub-optimal therapeutic response, alternative procedures such as therapeutic drug monitoring^[91]^ are used to quantify patient response on an individualized basis and to customize 5-FU treatment to account for individual variations in drug metabolism.

### Intratumor 5-FU catabolism

In addition to the role of *DPYD* polymorphisms in modulating 5-FU toxicity and therapeutic response by affecting systemic drug degradation, intra-tumoral *DPYD* expression is an important factor in modulating therapeutic response. For example, elevated intra-tumoral *DPYD* expression, together with elevated *TYMS*, is associated with poor outcomes in CRC patients treated with 5-FU-based chemotherapy^[66]^. A third gene, thymidine phosphorylase (TP; encoded by *TYMP*), was implicated together with *DPYD* and *TYMS* in this study. TP catalyzes a reversible reaction that may produce thymidine or 2’-deoxyuridine, or analogs such as 5-fluoro-2’-deoxyuridine (FdU), from their respective nucleobases (e.g., 5-FU), together with 2’-deoxyribose 1-phosphate. Alternatively, TP degrades thymidine analogs such as FdU to the nucleobase after dephosphorylation by ecto-5′-nucleotidase (NT5E)^[92]^. The directionality of TP catalysis depends on intra-tumor substrate/product ratios; however, levels of 2’-deoxyribose 1-phosphate in plasma were also found to be predictive of chemotherapy sensitivity in gastric cancer that included a fluoropyrimidine^[93]^. Findings from this study^[66]^ that elevated *TYMP* levels together with *TYMS* and *DPYD* are associated with decreased response to 5-FU are consistent with TP primarily catalyzing FdU degradation in CRC tumors and resistance to 5-FU is associated with elevated *TYMP* expression. Further, TP-mediated degradation of trifluorothymidine (TFT), the FP component of TAS-102, limits activity resulting in the inclusion of a TP inhibitor, Tipiracil^[94]^. TP is also known as platelet-derived endothelial cell growth factor (PDECGF), a growth factor promoting angiogenesis, and increased PDECGF/TP is a prognostic factor for poor survival in CRC^[95]^ that acts through the production of 2’-deoxyribose 1-phosphate from thymidine to promote chemotaxis of vascular endothelial cells^[96]^.

### Anabolic 5-FU metabolism and resistance

The anabolic biosynthesis of FdUMP from 5-FU can occur via either of two major pathways: (1) TP/thymidine kinase (TK) in which FdUMP is produced by 5-FU in two steps; or (2) via a multi-step biosynthetic pathway (UMPS/RNR) that involves UMP synthase (UMPS), uridine kinase (UK), and UMP kinase (UMPK) to produce FUDP. FUDP is a substrate for ribonucleotide reductase (RNR) to produce FdUDP, which can be converted to 5-fluoro-2’-deoxyuridine-5’-diphosphate (FdUDP) through conversion to FdUTP followed by dUTPase cleavage. Enzymes important for the *de novo* biosynthesis of pyrimidines are upregulated in CRC relative to non-malignant tissue^[97]^, and reduced activities of these enzymes which may occur via altered splicing^[88,98]^ are associated with 5-FU resistance^[99]^. The importance of FdUMP biosynthesis via the UMPS/RNR pathway is demonstrated by studies that identify reduced expression and activity of enzymes in this pathway in 5-FU-resistant cells. Studies in KM12C xenograft tumors showed resistance to 5-FU was associated with decreased RNR activity^[100]^, while analysis of clinical samples indicated 5-FU resistance was associated with high TS mRNA and low RNR activity^[101]^.

Collectively, the preponderance of evidence indicates that altered *de novo* thymidine biosynthesis, either by affecting TS expression [Figure 2] or modulating genes important for 5-FU anabolic metabolism to FdUMP [Figure 1], is central to 5-FU resistance. In a few instances, 5-FU resistance is mediated by changes affecting RNA-directed processes including tRNA modifications^[102,103]^ and rRNA^[22]^. However, the clinical significance of RNA-directed activities for 5-FU anti-tumor activity is not yet proven. Further evidence for anabolic metabolism of 5-FU to FdUMP being important for 5-FU resistance comes from studies demonstrating elevated expression of ABCC10^[104]^ and ABCC5^[105]^, two ATP binding cassette proteins^[106]^ that mediate FdUMP efflux from 5-FU-treated cells, cause of 5-FU resistance as does elevated FOXM1, a major transcriptional regulator of ABCC10^[104]^ [Figure 1].

## CELL DEATH SIGNALING IN 5-FU RESISTANCE

**Figure 3:**
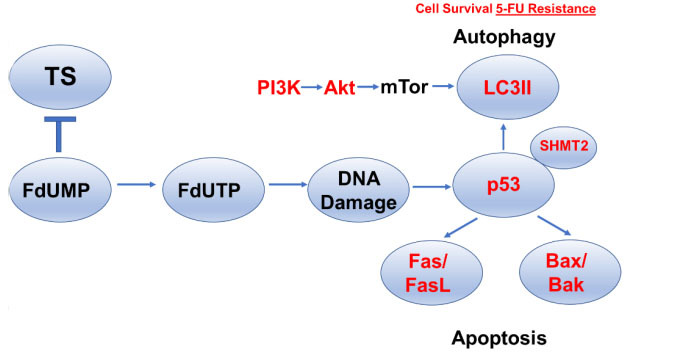
5-FU Resistance develops from an altered balance between autophagy, which favors cell survival and apoptosis in 5-FU-treated cells. p53 is a key regulator of autophagy/apoptosis balance in 5-FU-treated cells and is modulated by SHMT2. Increased signaling through the PI3K/Akt/mTOR pathway stimulates LC3II and upregulates autophagy to promote cell survival and 5-FU resistance. Induction of apoptosis involves upregulation of the death receptor pathway or mitochondrial pathway for programmed cell death, and downregulation of Fas/FasL or Bax/Bak decreases 5-FU-induced apoptosis and promotes cell survival and 5-FU resistance. 5-FU: 5-Fluorouracil; TS: thymidylate synthase; FdUMP: 5-Fluoro-2’-deoxyuridine-5’-O-monophosphate; FdUTP: 5-fluoro-2′-deoxyuridine-5′-triphosphate; SHMT2: serine hydroxymethyl transferase.

The cytotoxicity of multiple anti-cancer drugs, including 5-FU, depends on the activation of programmed cell death that irreversibly commits drug-treated cells to destruction^[107,108]^. p53 is considered to be the most highly mutated gene in cancer and it plays a central role in determining if drug-treated cells undergo cell cycle arrest mediated by p53’s downstream effector p21, or initiate apoptosis mediated by Bax and other p53-dependent pro-apoptotic genes^[109]^ [Figure 3]. In the case of established DNA-damaging drugs such as Adriamycin, either p53 or p21 deficiency leads to loss of the G1/S checkpoint and efficient apoptosis^[110]^. However, 5-FU deletion of p53 in HCT-116 cells resulted in resistance to apoptosis and 5-FU was less effective towards p53^-/-^ HCT-116 xenografts relative to isogenic tumors that were p53^+/+^. Furthermore, 5-FU-induced apoptosis both required p53 and was inhibited by exogenous uridine, but not thymidine, consistent with apoptosis induction in response to an RNA-directed process under these treatment conditions^[110]^. Studies from our laboratory confirm that p53 deletion causes 5-FU resistance in HCT-116 cells with expression of the R248W gain of function p53 mutation causing greater resistance, while the DNA-directed FP polymer CF10 showed reduced resistance indices relative to 5-FU^[1]^.

However, even in cell models of CRC, there is variability in the extent that p53 is required for 5-FU-induced apoptosis. Studies report that 5-FU-induced apoptosis occurs in both wild-type and mutant p53 CRC cells with increased expression of the pro-apoptotic Bcl-2 family proteins Bax and Bak identified as being particularly important for 5-FU-induced apoptosis^[111]^ [Figure 3]. The importance of p53 for regulating 5-FU-induced apoptosis has also been shown to occur via altered chromatin accessibility upon 5-FU treatment that affects the transcription of genes important for apoptosis^[112]^. The clinical significance of p53 mutations for 5-FU resistance is not established, although some clinical data indicate *TP53* mutations confer a worse prognosis^[113]^, while p53 together with Rb and the anti-apoptotic bcl-family member Mcl-1 correlated with clinical outcomes in patients with colorectal liver metastases^[114]^. Factors other than p53 that are important for activating apoptosis in response to 5-FU treatment include Fas, which was shown to be induced in response to 5-FU treatment in a p53-dependent manner and to regulate apoptosis^[115]^ [Figure 3]. Houghton and co-workers showed that different CRC cell lines varied in the efficiency of Fas upregulation in response to 5-FU/LV treatment and this correlated with the efficiency of thymidine reversal, indicating 5-FU’s DNA-directed effects were cell line dependent and correlated with Fas upregulation and activation of extrinsic apoptosis^[116]^. Resistance to 5-FU-induced Fas upregulation and apoptosis can occur via methylation of the Fas promoter, silencing its expression, which can be reversed by 5-Aza deoxycytidine^[117]^. Clinical significance for Fas upregulation in 5-FU response was shown by increased Fas expression in biopsy specimens following 5-FU treatment^[118]^.

## AUTOPHAGY IN 5-FU RESISTANCE

Autophagy plays an important role in tumorigenesis and modulating drug response and can either be complementary to apoptosis by promoting drug lethality or protective of the cytotoxic effects of drug treatment^[119]^. Activation of the PI3K/Akt/mTOR signaling pathway upregulates autophagosome formation and inhibitors of this pathway modulate drug response, in part, through downregulating autophagy. Treatment of CRC cells with 5-FU was shown to increase LC3-II levels consistent with autophagy activation and co-treatment with 3-methyl adenine (3-MA), a PI3K inhibitor, blocked autophagosome formation and promoted 5-FU-induced apoptosis implicating a protective role for autophagy in 5-FU treatment^[120]^ [Figure 3]. However, reduced autophagy has also been reported in 5-FU-resistant CRC cells^[121]^. Overactivation of the Akt pathway is also associated with 5-FU resistance and Akt inhibition may overcome resistance in some CRC cells^[122]^, in part by modulating autophagy, while miRNA regulation of Akt deactivation by the PP2A phosphatase complex also regulates 5-FU resistance^[123]^. The autophagy inhibitor chloroquine also enhanced the lethality of 5-FU to CRC cells, and in these studies, a serine hydroxymethyl transferase (SHMT2) was shown to regulate 5-FU resistance by binding p53 and inhibiting its degradation [Figure 3]. Overall, SHMT2 was upregulated in CRC tissue compared to non-malignant tissue; however, patients with low SHMT2 had worse outcomes and this correlated with elevated LC3-II and p62 consistent with autophagy activation in SHMT2-low, 5-FU-resistant CRC^[124]^. Interestingly, trifluorothymidine (TFT), the FP used in TAS-102, differed from 5-FU in the extent of activating autophagic survival^[125]^. Activation of the p38MAPK pathway is also a determinant in autophagy activation and modulates cellular responses to 5-FU. Inhibition of the p38MAPK pathway correlated with attenuation in 5-FU-mediated apoptosis and promoted CRC cell resistance^[126]^. Thus, the p38MAPK signaling pathway modulates 5-FU resistance by regulating the pivot between autophagy and apoptosis^[126]^. The autophagy-regulated gene HSPB8 was found to be key in regulating interactions with the tumor microenvironment that regulate 5-FU resistance^[127]^. Autophagosome formation is also regulated by Rho kinases^[128]^, which are implicated in 5-FU resistance^[129]^. Curcumin has been studied to inhibit AMPK/ULK1-dependent autophagy with the potential to overcome 5-FU resistance through autophagy activation^[130]^, and studies from our laboratory indicate curcumin enhances the cytotoxicity of DNA-directed polymeric fluoropyrimidines^[131,132]^.

## CONCLUSION

5-FU remains central to the management of colorectal cancer and it is widely used both in the adjuvant setting to treat CRC patients with limited-stage disease and in combination with chemotherapy regimens to treat mCRC^[4]^. The evasion of 5-FU cytotoxicity through intrinsic or acquired resistance in patient tumors contributes to poor outcomes manifest either as a high rate of relapse despite adjuvant chemotherapy or limited survival in the metastatic setting despite multiple lines of chemotherapy that include 5-FU or other FP drugs^[5]^. Lack of response to 5-FU chemotherapy is predictable in patients with deficiencies in DNA MMR, or high microsatellite instability, and therapy with 5-FU is contra-indicated in these patients. 5-FU therapy is also predictably toxic in patients with polymorphisms in *DPYD* that limit 5-FU degradation in the liver^[89]^, and these patients require special management^[90]^. Beyond these limited exclusions, there are currently no defined criteria for determining which CRC patients are not likely to be responsive to 5-FU-based therapy. Thus, there is a need to systematically understand the mechanistic basis for 5-FU treatment failure, and an urgent need to develop new approaches for circumventing major causes of 5-FU resistance.

In this review, we have summarized major mechanisms that contribute to 5-FU resistance with an emphasis on those for which available data support clinical significance and that affect the on-target activity of 5-FU (TS inhibition). The causes of colorectal cancer are multi-factorial and involve both lifestyle choice and personalized genetic susceptibility^[133]^. Further, response to treatment also depends on multiple factors^[134]^. Collectively, the reviewed literature consistently implicates resistance as developing from processes that limit the anabolic metabolism of 5-FU to FdUMP, the TS inhibitory metabolite [Figure 1], and from mechanisms that result in elevated TS activity that results from gene amplification, polymorphisms in the TS promoter, elevated levels of transcription factors that regulate *TYMS* expression, and/or altered nuclear localization of TS [Figure 2]. Dysregulation in the balance between cell survival and programmed cell death is also important in the development of 5-FU resistance [Figure 3]. We have not attempted to review miRNA regulation of *TYMS* ^[61]^ or other epigenetic causes of 5-FU resistance^[135]^, and these have recently been reviewed^[136-138]^. We also have not directed the reader to literature focused on cellular changes that promote quiescence and stemness to escape the cytotoxic effects of 5-FU^[139,140]^, although these are likely to be of clinical significance. Further, it is clear that the tumor microenvironment modulates therapy response by processes independent of on-target effects on cancer cells and these processes are not reviewed in this manuscript.

The focus of this review is on acquired resistance to 5-FU with decreased anabolic metabolism and elevated TS activity^[49]^ as clinically relevant causes. In principle, these causes of 5-FU can be addressed through the translation of next-generation FP drugs that retain the anti-tumor activity associated with targeting TS in CRC, but that do not require multiple steps of anabolic metabolism required by 5-FU. TAS-102, a combination of the FP trifluorothymidine and a TP inhibitor Tiperacil, shows efficacy in 5-FU-resistant models and activity in refractory metastatic CRC^[141]^. TAS-102 activation requires thymidine kinase and it is not a substrate for DPD^[94]^. Our laboratory has pioneered the development of DNA-based FP polymers to deliver Fluorodeoxyuridylate, the TS-inhibitory metabolite of 5-FU, without a requirement for metabolic activation. We showed that the prototype FP polymer F10 was, on average, 338-fold more potent than 5-FU across the NCI60 cell line screen^[15,135]^, but it was still very well tolerated *in vivo*^[142]^, indicating 5-FU toxicities do not necessarily arise predominantly from on-target effects. The 2^nd^ generation FP polymer CF10 is even more potent and shows promising activity in pre-clinical models of CRC and pancreatic cancer^[16,17]^. Further, the cytotoxic mechanism of CF10 results from both inhibiting TS and poisoning of DNA topoisomerase 1 (Top1)^[41,143]^, which results from a distinct mechanism distinct from current Top1 poisons in clinical use^[144]^. In summary, the next generation of FPs has the potential to overcome the established mechanism of resistance for 5-FU reviewed herein that has limited clinical response to FPs to date.

## DECLARATIONS

### Copyright

© The Author(s) 2023.

## References

[1] Dominijanni A, Gmeiner WH (2018). Improved potency of F10 relative to 5-fluorouracil in colorectal cancer cells with p53 mutations. Cancer Drug Resist.

[2] Bray F, Ferlay J, Soerjomataram I, Siegel RL, Torre LA, Jemal A (2018). Global cancer statistics 2018: GLOBOCAN estimates of incidence and mortality worldwide for 36 cancers in 185 countries. CA Cancer J Clin.

[3] González-Senac NM, Mayordomo-Cava J, Macías-Valle A (2021). Colorectal cancer in elderly patients with surgical indication: state of the art, current management, role of frailty and benefits of a geriatric liaison. Int J Environ Res Public Health.

[4] Gmeiner WH (2021). Recent advances in our knowledge of mCRC tumor biology and genetics: a focus on targeted therapy development. Onco Targets Ther.

[5] Punt CJ, Koopman M, Vermeulen L (2017). From tumour heterogeneity to advances in precision treatment of colorectal cancer. Nat Rev Clin Oncol.

[6] Sethy C, Kundu CN (2021). 5-Fluorouracil (5-FU) resistance and the new strategy to enhance the sensitivity against cancer: Implication of DNA repair inhibition. Biomed Pharmacother.

[7] Długosz-Pokorska A, Pięta M, Janecki T, Janecka A (2019). New uracil analogs as downregulators of ABC transporters in 5-fluorouracil-resistant human leukemia HL-60 cell line. Mol Biol Rep.

[8] Chen P, Ni W, Xie T, Sui X (2019). Meta-Analysis of 5-Fluorouracil-Based Chemotherapy Combined With Traditional Chinese Medicines for Colorectal Cancer Treatment. Integr Cancer Ther.

[9] Luqmani YA (2005). Mechanisms of drug resistance in cancer chemotherapy. Med Princ Pract.

[10] Morawska K, Goirand F, Marceau L (2018). 5-FU therapeutic drug monitoring as a valuable option to reduce toxicity in patients with gastrointestinal cancer. Oncotarget.

[11] Weckbecker G (1991). Biochemical pharmacology and analysis of fluoropyrimidines alone and in combination with modulators. Pharmacol Ther.

[12] Carreras CW, Naber N, Cooke R, Santi DV (1994). A C-terminal conformational equilibrium in thymidylate synthase observed by electron paramagnetic resonance spectroscopy. Biochemistry.

[13] Wilson PM, Danenberg PV, Johnston PG, Lenz HJ, Ladner RD (2014). Standing the test of time: targeting thymidylate biosynthesis in cancer therapy. Nat Rev Clin Oncol.

[14] Luengo A, Gui DY, Vander Heiden MG (2017). Targeting metabolism for cancer therapy. Cell Chem Biol.

[15] Liao ZY, Sordet O, Zhang HL (2005). A novel polypyrimidine antitumor agent FdUMP[10] induces thymineless death with topoisomerase I-DNA complexes. Cancer Res.

[16] Gmeiner WH, Dominijanni A, Haber AO (2021). Improved antitumor activity of the Fluoropyrimidine polymer CF10 in preclinical colorectal cancer models through distinct mechanistic and pharmacologic properties. Mol Cancer Ther.

[17] Haber AO, Jain A, Mani C (2021). AraC-FdUMP[10] Is a next-generation Fluoropyrimidine with potent antitumor activity in PDAC and synergy with PARG inhibition. Mol Cancer Res.

[18] Mani C, Pai S, Papke CM, Palle K, Gmeiner WH (2018). Thymineless death by the fluoropyrimidine polymer F10 involves replication fork collapse and is inhanced by Chk1 inhibition. Neoplasia.

[19] Miura K, Kinouchi M, Ishida K (2010). 5-fu metabolism in cancer and orally-administrable 5-fu drugs. Cancers (Basel).

[20] Diasio RB, Harris BE (1989). Clinical pharmacology of 5-fluorouracil. Clin Pharmacokinet.

[21] Longley DB, Harkin DP, Johnston PG (2003). 5-fluorouracil: mechanisms of action and clinical strategies. Nat Rev Cancer.

[22] Chalabi-Dchar M, Fenouil T, Machon C (2021). A novel view on an old drug, 5-fluorouracil: an unexpected RNA modifier with intriguing impact on cancer cell fate. NAR Cancer.

[23] Gmeiner WH (2020). Fluoropyrimidine Modulation of the Anti-Tumor Immune Response-Prospects for Improved Colorectal Cancer Treatment. Cancers (Basel).

[24] Pritchard DM, Watson AJ, Potten CS, Jackman AL, Hickman JA (1997). Inhibition by uridine but not thymidine of p53-dependent intestinal apoptosis initiated by 5-fluorouracil: evidence for the involvement of RNA perturbation. Proc Natl Acad Sci U S A.

[25] (1989). Groeningen CJ, Peters GJ, Leyva A, Laurensse E, Pinedo HM. Reversal of 5-fluorouracil-induced myelosuppression by prolonged administration of high-dose uridine. J Natl Cancer Inst.

[26] Saif MW, Diasio RB (2016). Benefit of uridine triacetate (Vistogard) in rescuing severe 5-fluorouracil toxicity in patients with dihydropyrimidine dehydrogenase (DPYD) deficiency. Cancer Chemother Pharmacol.

[27] Saif MW, Hachem H, Purvey S (2020). Pharmacogenetic variants in the DPYD and TYMS genes are clinically significant predictors of fluoropyrimidine toxicity: are we ready for use in our clinical practice. Arch Pharmacol Ther.

[28] Saif MW, Syrigos K, Mehra R, Mattison LK, Diasio RB (2007). Dihydropyrimidine dehydrogenase deficiency (Dpd) in Gi malignancies: experience of 4-years. Pak J Med Sci.

[29] Botrel TEA, Clark LGO, Paladini L, Clark OAC (2016). Efficacy and safety of bevacizumab plus chemotherapy compared to chemotherapy alone in previously untreated advanced or metastatic colorectal cancer: a systematic review and meta-analysis. BMC Cancer.

[30] Liu Z, Xu Y, Xu G (2021). Nomogram for predicting overall survival in colorectal cancer with distant metastasis. BMC Gastroenterol.

[31] Veenstra CM, Krauss JC (2018). Emerging systemic therapies for colorectal cancer. Clin Colon Rectal Surg.

[32] Shen C, Tannenbaum D, Horn R (2022). Overall survival in phase 3 clinical trials and the surveillance, epidemiology, and end results database in patients with metastatic colorectal cancer, 1986-2016: a systematic review. JAMA Netw Open.

[33] André T, Cohen R, Salem ME (2022). Immune checkpoint blockade therapy in patients with colorectal cancer harboring microsatellite instability/mismatch repair deficiency in 2022. Am Soc Clin Oncol Educ Book.

[34] Adeleke S, Haslam A, Choy A (2022). Microsatellite instability testing in colorectal patients with Lynch syndrome: lessons learned from a case report and how to avoid such pitfalls. Per Med.

[35] Aiyer KTS, Doeleman T, Ryan NA (2022). Validity of a two-antibody testing algorithm for mismatch repair deficiency testing in cancer; a systematic literature review and meta-analysis. Mod Pathol.

[36] Tougeron D, Mouillet G, Trouilloud I (2016). Efficacy of adjuvant chemotherapy in colon cancer with microsatellite instability: a large multicenter AGEO study. J Natl Cancer Inst.

[37] Watanabe T, Wu TT, Catalano PJ (2001). Molecular predictors of survival after adjuvant chemotherapy for colon cancer. N Engl J Med.

[38] Romano G, Santi L, Bianco MR (2016). The TGF-β pathway is activated by 5-fluorouracil treatment in drug resistant colorectal carcinoma cells. Oncotarget.

[39] Jung G, Benítez-Ribas D, Sánchez A, Balaguer F (2020). Current treatments of metastatic colorectal cancer with immune checkpoint inhibitors-2020 update. J Clin Med.

[40] Carethers JM, Chauhan DP, Fink D (1999). Mismatch repair proficiency and in vitro response to 5-fluorouracil. Gastroenterology.

[41] Gmeiner WH (2019). Entrapment of DNA topoisomerase-DNA complexes by nucleotide/nucleoside analogs. Cancer Drug Resist.

[42] Li LS, Morales JC, Veigl M (2009). DNA mismatch repair (MMR)-dependent 5-fluorouracil cytotoxicity and the potential for new therapeutic targets. Br J Pharmacol.

[43] Gmeiner WH, Mani C, Palle K Abstract 2828: MMR status affects efficiency of homologous recombination repair of F10-induced DNA DSBs. Cancer Res.

[44] Wyatt MD, Wilson DM 3rd (2009). Participation of DNA repair in the response to 5-fluorouracil. Cell Mol Life Sci.

[45] An Q, Robins P, Lindahl T, Barnes DE (2007). 5-Fluorouracil incorporated into DNA is excised by the Smug1 DNA glycosylase to reduce drug cytotoxicity. Cancer Res.

[46] Bracht K, Nicholls AM, Liu Y, Bodmer WF (2010). 5-Fluorouracil response in a large panel of colorectal cancer cell lines is associated with mismatch repair deficiency. Br J Cancer.

[47] Malki A, ElRuz RA, Gupta I, Allouch A, Vranic S, Al Moustafa AE (2020). Molecular mechanisms of colon cancer progression and metastasis: recent insights and advancements. Int J Mol Sci.

[48] Cheng YW, Pincas H, Bacolod MD (2008). CpG island methylator phenotype associates with low-degree chromosomal abnormalities in colorectal cancer. Clin Cancer Res.

[49] Gmeiner WH

[50] Yu J, Gao Y, Chen L (2022). Effect of S-1 plus Oxaliplatin compared with Fluorouracil, Leucovorin plus Oxaliplatin as perioperative chemotherapy for locally advanced, resectable gastric cancer: a randomized clinical trial. JAMA Netw Open.

[51] Tong D, Wang L, Mendis J, Essapen S (2021). Long term real-world outcomes of Trifluridine/Tipiracil in metastatic colorectal cancer-a single UK centre experience. Curr Oncol.

[52] Peters GJ, van der Wilt CL, van Groeningen CJ, Smid K, Meijer S, Pinedo HM (1994). Thymidylate synthase inhibition after administration of fluorouracil with or without leucovorin in colon cancer patients: implications for treatment with fluorouracil. J Clin Oncol.

[53] Noordhuis P, Holwerda U, Van der Wilt CL (2004). 5-Fluorouracil incorporation into RNA and DNA in relation to thymidylate synthase inhibition of human colorectal cancers. Ann Oncol.

[54] Kasahara M, Takahashi Y, Nagata T (2000). Thymidylate synthase expression correlates closely with E2F1 expression in colon cancer. Clin Cancer Res.

[55] Fang Z, Lin M, Li C, Liu H, Gong C (2020). A comprehensive review of the roles of E2F1 in colon cancer. Am J Cancer Res.

[56] Mannava S, Grachtchouk V, Wheeler LJ (2008). Direct role of nucleotide metabolism in C-MYC-dependent proliferation of melanoma cells. Cell Cycle.

[57] Drubin DA, Hess AK, Catlett NL, Cara AD, Wettergren Y, Tell R (2021). MYC as a candidate upstream controller involved in TYMS gene expression and 5-FU/folate treatment efficacy in colorectal cancer. Journa of Clinical Oncology.

[58] Varghese V, Magnani L, Harada-Shoji N (2019). FOXM1 modulates 5-FU resistance in colorectal cancer through regulating TYMS expression. Sci Rep.

[59] Klinhom-On N, Seubwai W, Sawanyawisuth K, Obchoei S, Mahalapbutr P, Wongkham S (2021). FOXM1 inhibitor, Siomycin A, synergizes and restores 5-FU cytotoxicity in human cholangiocarcinoma cell lines via targeting thymidylate synthase. Life Sci.

[60] Ahn JY, Lee JS, Min HY, Lee HY (2015). Acquired resistance to 5-fluorouracil via HSP90/Src-mediated increase in thymidylate synthase expression in colon cancer. Oncotarget.

[61] Matuszyk J (2022). MALAT1-miRNAs network regulate thymidylate synthase and affect 5FU-based chemotherapy. Mol Med.

[62] Zhang Y, Wang J (2017). MicroRNAs are important regulators of drug resistance in colorectal cancer. Biol Chem.

[63] Ogunwobi OO, Mahmood F, Akingboye A (2020). Biomarkers in colorectal cancer: current research and future prospects. Int J Mol Sci.

[64] Johnston PG, Lenz HJ, Leichman CG (1995). Thymidylate synthase gene and protein expression correlate and are associated with response to 5-fluorouracil in human colorectal and gastric tumors. Cancer Res.

[65] Copur S, Aiba K, Drake JC, Allegra CJ, Chu E (1995). Thymidylate synthase gene amplification in human colon cancer cell lines resistant to 5-fluorouracil. Biochem Pharmacol.

[66] Salonga D, Danenberg KD, Johnson M (2000). Colorectal tumors responding to 5-fluorouracil have low gene expression levels of dihydropyrimidine dehydrogenase, thymidylate synthase, and thymidine phosphorylase. Clin Cancer Res.

[67] Edler D, Kressner U, Ragnhammar P (2000). Immunohistochemically detected thymidylate synthase in colorectal cancer: an independent prognostic factor of survival. Clin Cancer Res.

[68] Edler D, Glimelius B, Hallström M (2002). Thymidylate synthase expression in colorectal cancer: a prognostic and predictive marker of benefit from adjuvant fluorouracil-based chemotherapy. J Clin Oncol.

[69] Popat S, Matakidou A, Houlston RS (2004). Thymidylate synthase expression and prognosis in colorectal cancer: a systematic review and meta-analysis. J Clin Oncol.

[70] Kamynina E, Stover PJ

[71] Chon J, Stover PJ, Field MS (2017). Targeting nuclear thymidylate biosynthesis. Mol Aspects Med.

[72] Gustavson MD, Molinaro AM, Tedeschi G, Camp RL, Rimm DL (2008). AQUA analysis of thymidylate synthase reveals localization to be a key prognostic biomarker in 2 large cohorts of colorectal carcinoma. Arch Pathol Lab Med.

[73] Watson RG, Muhale F, Thorne LB (2010). Amplification of thymidylate synthetase in metastatic colorectal cancer patients pretreated with 5-fluorouracil-based chemotherapy. Eur J Cancer.

[74] Wang TL, Diaz LA Jr, Romans K (2004). Digital karyotyping identifies thymidylate synthase amplification as a mechanism of resistance to 5-fluorouracil in metastatic colorectal cancer patients. Proc Natl Acad Sci U S A.

[75] Clark JL, Berger SH, Mittelman A, Berger FG (1987). Thymidylate synthase gene amplification in a colon tumor resistant to fluoropyrimidine chemotherapy. Cancer Treat Rep.

[76] Berger SH, Jenh CH, Johnson LF, Berger FG (1985). Thymidylate synthase overproduction and gene amplification in fluorodeoxyuridine-resistant human cells. Mol Pharmacol.

[77] Marsh S, McKay JA, Cassidy J, McLeod HL (2001). Polymorphism in the thymidylate synthase promoter enhancer region in colorectal cancer. Int J Oncol.

[78] Grumetti L, Lombardi R, Iannelli F (2022). Epigenetic approaches to overcome Fluoropyrimidines resistance in solid tumors. Cancers (Basel).

[79] Wang YC, Xue HP, Wang ZH, Fang JY (2013). An integrated analysis of the association between Ts gene polymorphisms and clinical outcome in gastric and colorectal cancer patients treated with 5-FU-based regimens. Mol Biol Rep.

[80] Mauritz R, Giovannetti E, Beumer IJ (2009). Polymorphisms in the enhancer region of the thymidylate synthase gene are associated with thymidylate synthase levels in normal tissues but not in malignant tissues of patients with colorectal cancer. Clin Colorectal Cancer.

[81] Peters GJ, Backus HH, Freemantle S (2002). Induction of thymidylate synthase as a 5-fluorouracil resistance mechanism. Biochim Biophys Acta.

[82] Goff LW, Thakkar N, Du L (2014). Thymidylate synthase genotype-directed chemotherapy for patients with gastric and gastroesophageal junction cancers. PLoS One.

[83] Ntavatzikos A, Spathis A, Patapis P (2019). TYMS/KRAS/BRAF molecular profiling predicts survival following adjuvant chemotherapy in colorectal cancer. World J Gastrointest Oncol.

[84] Therizols G, Bash-Imam Z, Panthu B (2022). Alteration of ribosome function upon 5-fluorouracil treatment favors cancer cell drug-tolerance. Nat Commun.

[85] Very N, Hardivillé S, Decourcelle A (2022). Thymidylate synthase O-GlcNAcylation: a molecular mechanism of 5-FU sensitization in colorectal cancer. Oncogene.

[86] Zhang N, Yin Y, Xu SJ, Chen WS (2008). 5-Fluorouracil: mechanisms of resistance and reversal strategies. Molecules.

[87] Matuo R, Sousa FG, Escargueil AE (2009). 5-Fluorouracil and its active metabolite FdUMP cause DNA damage in human SW620 colon adenocarcinoma cell line. J Appl Toxicol.

[88] Humeniuk R, Menon LG, Mishra PJ (2009). Decreased levels of UMP kinase as a mechanism of fluoropyrimidine resistance. Mol Cancer Ther.

[89] Diasio RB, Offer SM (2022). Testing for Dihydropyrimidine Dehydrogenase deficiency to individualize 5-Fluorouracil therapy. Cancers (Basel).

[90] Gmeiner WH (2021). A narrative review of genetic factors affecting fluoropyrimidine toxicity. Precis Cancer Med.

[91] Lee JJ, Beumer JH, Chu E (2016). Therapeutic drug monitoring of 5-fluorouracil. Cancer Chemother Pharmacol.

[92] Hu S, Meng F, Yin X, Cao C, Zhang G (2019). NT5E is associated with unfavorable prognosis and regulates cell proliferation and motility in gastric cancer. Biosci Rep.

[93] Wang D, Li W, Yin L, Du Y, Zhang S, Suo J (2020). Association of serum levels of deoxyribose 1-phosphate and S-lactoylglutathione with neoadjuvant chemotherapy sensitivity in patients with gastric cancer: A metabolomics study. Oncol Lett.

[94] Peters GJ (2015). Therapeutic potential of TAS-102 in the treatment of gastrointestinal malignancies. Ther Adv Med Oncol.

[95] Takebayashi Y, Akiyama S, Akiba S (1996). Clinicopathologic and prognostic significance of an angiogenic factor, thymidine phosphorylase, in human colorectal carcinoma. J Natl Cancer Inst.

[96] Bijnsdorp IV, Azijli K, Jansen EE (2010). Accumulation of thymidine-derived sugars in thymidine phosphorylase overexpressing cells. Biochem Pharmacol.

[97] Matsusaka S, Yamasaki H, Fukushima M, Wakabayashi I (2007). Upregulation of enzymes metabolizing 5-fluorouracil in colorectal cancer. Chemotherapy.

[98] Griffith M, Mwenifumbo JC, Cheung PY (2013). Novel mRNA isoforms and mutations of uridine monophosphate synthetase and 5-fluorouracil resistance in colorectal cancer. Pharmacogenomics J.

[99] Fujii R, Seshimo A, Kameoka S (2003). Relationships between the expression of thymidylate synthase, dihydropyrimidine dehydrogenase, and orotate phosphoribosyltransferase and cell proliferative activity and 5-fluorouracil sensitivity in colorectal carcinoma. Int J Clin Oncol.

[100] Fukushima M, Fujioka A, Uchida J, Nakagawa F, Takechi T (2001). Thymidylate synthase (TS) and ribonucleotide reductase (RNR) may be involved in acquired resistance to 5-fluorouracil (5-FU) in human cancer xenografts in vivo. Eur J Cancer.

[101] Kubota T, Watanabe M, Otani Y, Kitajima M, Fukushiuma M (2002). Different pathways of 5-fluorouracil metabolism after continuous venous or bolus injection in patients with colon carcinoma: possible predictive value of thymidylate synthetase mRNA and ribonucleotide reductase for 5-fluorouracil sensitivity. Anticancer Res.

[102] Gustavsson M, Ronne H (2008). Evidence that tRNA modifying enzymes are important in vivo targets for 5-fluorouracil in yeast. RNA.

[103] Okamoto M, Fujiwara M, Hori M (2014). tRNA modifying enzymes, NSUN2 and METTL1, determine sensitivity to 5-fluorouracil in HeLa cells. PLoS Genet.

[104] Xie T, Geng J, Wang Y (2017). FOXM1 evokes 5-fluorouracil resistance in colorectal cancer depending on ABCC10. Oncotarget.

[105] Pratt S, Shepard RL, Kandasamy RA, Johnston PA, Perry W 3rd, Dantzig AH (2005). The multidrug resistance protein 5 (ABCC5) confers resistance to 5-fluorouracil and transports its monophosphorylated metabolites. Mol Cancer Ther.

[106] Marin JJG, Monte MJ, Macias RIR (2022). Expression of Chemoresistance-Associated ABC Proteins in Hepatobiliary, Pancreatic and Gastrointestinal Cancers. Cancers (Basel).

[107] Hientz K, Mohr A, Bhakta-Guha D, Efferth T (2017). The role of p53 in cancer drug resistance and targeted chemotherapy. Oncotarget.

[108] Fulda S, Debatin KM (2006). Extrinsic versus intrinsic apoptosis pathways in anticancer chemotherapy. Oncogene.

[109] Bunz F, Dutriaux A, Lengauer C (1998). Requirement for p53 and p21 to sustain G2 arrest after DNA damage. Science.

[110] Bunz F, Hwang PM, Torrance C (1999). Disruption of p53 in human cancer cells alters the responses to therapeutic agents. J Clin Invest.

[111] Nita ME, Nagawa H, Tominaga O (1998). 5-Fluorouracil induces apoptosis in human colon cancer cell lines with modulation of Bcl-2 family proteins. Br J Cancer.

[112] Yang CM, Kang MK, Jung WJ (2021). p53 expression confers sensitivity to 5-fluorouracil via distinct chromatin accessibility dynamics in human colorectal cancer. Oncol Lett.

[113] Kandioler D, Mittlböck M, Kappel S, p53 Research Group and the Austrian Breast and Colorectal Study Group (ABCSG) (2015). TP53 mutational status and prediction of benefit from adjuvant 5-Fluorouracil in stage III colon cancer patients. EBioMedicine.

[114] Backus HH, van Riel JM, van Groeningen CJ (2001). Rb, mcl-1 and p53 expression correlate with clinical outcome in patients with liver metastases from colorectal cancer. Ann Oncol.

[115] Longley DB, Allen WL, McDermott U (2004). The roles of thymidylate synthase and p53 in regulating Fas-mediated apoptosis in response to antimetabolites. Clin Cancer Res.

[116] Tillman DM, Petak I, Houghton JA (1999). A Fas-dependent component in 5-fluorouracil/leucovorin-induced cytotoxicity in colon carcinoma cells. Clin Cancer Res.

[117] Petak I, Danam RP, Tillman DM (2003). Hypermethylation of the gene promoter and enhancer region can regulate Fas expression and sensitivity in colon carcinoma. Cell Death Differ.

[118] Backus HH, Dukers DF, van Groeningen CJ (2001). 5-Fluorouracil induced Fas upregulation associated with apoptosis in liver metastases of colorectal cancer patients. Ann Oncol.

[119] Xie Q, Liu Y, Li X (2020). The interaction mechanism between autophagy and apoptosis in colon cancer. Transl Oncol.

[120] Li J, Hou N, Faried A, Tsutsumi S, Kuwano H (2010). Inhibition of autophagy augments 5-fluorouracil chemotherapy in human colon cancer in vitro and in vivo model. Eur J Cancer.

[121] Yao CW, Kang KA, Piao MJ (2017). Reduced autophagy in 5-Fluorouracil resistant colon cancer cells. Biomol Ther (Seoul).

[122] Kim EJ, Kang GJ, Kang JI (2018). Over-activation of AKT signaling leading to 5-Fluorouracil resistance in SNU-C5/5-FU cells. Oncotarget.

[123] Zhang Y, Talmon G, Wang J (2015). MicroRNA-587 antagonizes 5-FU-induced apoptosis and confers drug resistance by regulating PPP2R1B expression in colorectal cancer. Cell Death Dis.

[124] Chen J, Na R, Xiao C (2021). The loss of SHMT2 mediates 5-fluorouracil chemoresistance in colorectal cancer by upregulating autophagy. Oncogene.

[125] Bijnsdorp IV, Peters GJ, Temmink OH, Fukushima M, Kruyt FA (2010). Differential activation of cell death and autophagy results in an increased cytotoxic potential for trifluorothymidine compared to 5-fluorouracil in colon cancer cells. Int J Cancer.

[126] de la Cruz-Morcillo MA, Valero ML, Callejas-Valera JL (2012). P38MAPK is a major determinant of the balance between apoptosis and autophagy triggered by 5-fluorouracil: implication in resistance. Oncogene.

[127] Gao T, Yuan D, He B (2022). Identification of autophagy related genes in predicting the prognosis and aiding 5- fluorouracil therapy of colorectal cancer. Heliyon.

[128] Mleczak A, Millar S, Tooze SA, Olson MF, Chan EY (2013). Regulation of autophagosome formation by Rho kinase. Cell Signal.

[129] Zheng Z, He XY, Li JF (2013). RhoGDI2 confers resistance to 5-fluorouracil in human gastric cancer cells. Oncol Lett.

[130] Zhang P, Lai ZL, Chen HF (2017). Curcumin synergizes with 5-fluorouracil by impairing AMPK/ULK1-dependent autophagy, AKT activity and enhancing apoptosis in colon cancer cells with tumor growth inhibition in xenograft mice. J Exp Clin Cancer Res.

[131] Ghosh S, Mallick S, Das U (2018). Curcumin stably interacts with DNA hairpin through minor groove binding and demonstrates enhanced cytotoxicity in combination with FdU nucleotides. Biochim Biophys Acta Gen Subj.

[132] Das U, Bhuniya A, Roy AK, Gmeiner WH, Ghosh S (2020). Hairpin Oligonucleotide can functionalize gold nanorods for in vivo application delivering cytotoxic nucleotides and curcumin: a comprehensive study in combination with near-infrared laser. ACS Omega.

[133] Anand P, Kunnumakkara AB, Sundaram C (2008). Cancer is a preventable disease that requires major lifestyle changes. Pharm Res.

[134] Azwar S, Seow HF, Abdullah M, Faisal Jabar M, Mohtarrudin N (2021). Recent updates on mechanisms of resistance to 5-Fluorouracil and reversal strategies in colon cancer treatment. Biology (Basel).

[135] Gmeiner WH, Reinhold WC, Pommier Y (2010). Genome-wide mRNA and microRNA profiling of the NCI 60 cell-line screen and comparison of FdUMP[10] with fluorouracil, floxuridine, and topoisomerase 1 poisons. Mol Cancer Ther.

[136] Deng J, Wang Y, Lei J, Lei W, Xiong JP (2017). Insights into the involvement of noncoding RNAs in 5-fluorouracil drug resistance. Tumour Biol.

[137] Marjaneh RM, Khazaei M, Ferns GA, Avan A, Aghaee-Bakhtiari SH (2019). The role of microRNAs in 5-FU resistance of colorectal cancer: possible mechanisms. J Cell Physiol.

[138] Ghafouri-Fard S, Abak A, Tondro Anamag F (2021). 5-Fluorouracil: a narrative review on the role of regulatory mechanisms in driving resistance to this chemotherapeutic agent. Front Oncol.

[139] Touil Y, Igoudjil W, Corvaisier M (2014). Colon cancer cells escape 5FU chemotherapy-induced cell death by entering stemness and quiescence associated with the c-Yes/YAP axis. Clin Cancer Res.

[140] Cho YH, Ro EJ, Yoon JS (2020). 5-FU promotes stemness of colorectal cancer via p53-mediated WNT/β-catenin pathway activation. Nat Commun.

[141] Siebenhüner A, De Dosso S, Meisel A, Wagner AD, Borner M (2020). Metastatic colorectal carcinoma after second progression and the role of Trifluridine-Tipiracil (TAS-102) in Switzerland. Oncol Res Treat.

[142] Pardee TS, Gomes E, Jennings-Gee J, Caudell D, Gmeiner WH (2012). Unique dual targeting of thymidylate synthase and topoisomerase1 by FdUMP[10] results in high efficacy against AML and low toxicity. Blood.

[143] Gmeiner WH, van Waardenburg RCAM (2021). Targeting DNA topoisomerases: past & future. Cancer Drug Resist.

[144] Pommier Y (2013). Drugging topoisomerases: lessons and challenges. ACS Chem Biol.

